# Diversity Analysis of Leaf Nutrient Endophytes and Metabolites in Dioecious *Idesia polycarpa* Maxim Leaves during Reproductive Stages

**DOI:** 10.3390/life12122041

**Published:** 2022-12-06

**Authors:** Jian Feng, Sohel Rana, Zhen Liu, Yanmei Wang, Qifei Cai, Xiaodong Geng, Huina Zhou, Tao Zhang, Shasha Wang, Xiaoyan Xue, Mingwan Li, Razia Sultana Jemim, Zhi Li

**Affiliations:** 1 College of Forestry, Henan Agricultural University, Zhengzhou 450046, China; 2 College of Life Sciences, Henan Agricultural University, Zhengzhou 450046, China

**Keywords:** dioecious plant, reproductive stages, nutrient characteristics, endophytes, metabolite

## Abstract

Leaves are essential vegetative organs of plants. Studying the variations in leaf nutrient content and microbial communities of male and female plants at reproductive stages helps us understand allocation and adaptation strategies. This study aimed to determine the nutrient characteristics and microbial differences in the leaves of male and female *Idesia polycarpa* at reproductive stages. Seven-year-old female and male plants were used as test materials in this experiment. The samples were collected at three stages: flowering (May), fruit matter accumulation (July), and fruit ripening (October). The nitrogen (TN), phosphorus (TP), potassium (TK), carbon (TC), and the pH of the female and male leaves were analyzed. In addition, the leaf microbial diversity and differential metabolites were determined using the Illumina high-throughput sequencing method and the ultra-high performance liquid chromatography–tandem mass spectrometry (UPLC–MS/MS) method at the reproductive developmental stages. This study found that male and female plant leaves had different TN and TK contents over time but no difference in TC and TP content. The significant differences in bacterial diversity between male and female plants and the richness of the fungi of male plants at the flowering and fruit maturity stages were observed. Proteobacteria, Pseudomonadaceae, Ascomycota, and Aspergillus were the dominant bacteria and fungi in the *Idesia polycarpa* leaves. The presence of microorganisms differed in the two sexes in different periods. Alphaproteobacteria and Sordariomycetes were the indicator groups for male leaves, and Pseudomonas and Sordariomycetes were the indicator groups for female leaves. Significant differences in phenolic acid were found between male and female leaves. A KEGG enrichment analysis revealed that differential metabolites were enriched in metabolic pathways, amino acid biosynthesis, and the nucleotide metabolism. According to a correlation analysis, leaf TK and TP were strongly correlated with endophytic bacteria abundance and differential metabolite composition. This study revealed the changes in substances and microorganisms in the leaves of male and female plants in their reproductive stages. It provides a theoretical basis for developing and utilizing the leaves of *Idesia polycarpa* and for field management.

## 1. Introduction

Plants, including dioecious plants, gradually evolved various breeding systems to adapt to their natural environments. Dioecious plants show significant differences in their morphological characteristics, physiological and biochemical responses, and gene expression due to their different reproductive costs [1]. The length–width ratio of females in *Rhus typhina* is significantly higher than that of males [2]. Another study showed that the growth of females exceeded that of males in terms of length extension, diameter growth, and leaf production [3]. Additionally, in the leaves of *Excoecaria agallocha*, total carotenoids, phenolic compounds, and protein concentrations were higher in female plants [4]. The oil palm orthologs of acid phosphatase and deficiency showed male-specific expression patterns [5]. Resource and growth microenvironment distribution between male and female plants are significantly different.

Leaves are one of the main organs by which plants obtain and utilize resources. Changes in the microenvironment of leaves can reflect the adaptation strategies of plants to different environments [6]. The nutrient content in leaves can indicate the plant growth status and habitat conditions. For example, nitrogen is an essential component of various key cellular molecules such as proteins, nucleic acids, and secondary metabolites, which are important for maintaining plant metabolism and growth [7]. Previous studies have shown a significant relationship between leaf nutrient content and gender. For example, the effective nitrogen content of female leaves of *Fraxinus mandshurica*, *Populus davidiana Dode*, and *Taxus cuspidata* was higher than that of male leaves [8]. The content of nutrient elements in the leaves of male *Fraxinus velutina* Torr plants was higher than that of female plants at each stage of the growing season [9]. Therefore, it is important to clarify the dynamic changes in leaf nutrients between dioecious plants for forest land management. In addition, many microbial communities in plant leaves interact and coevolve.

Studies have found that some endophytic microorganisms perform various important biological functions. For example, Zuo et al. [10] have confirmed that *Bacillus velezensis* BHZ-29 in Shihezi cotton plants can increase the activity of defense-related enzymes in cotton to varying degrees, as well as reduce the accumulation of MDA and thus enhance the resistance of cotton plants to diseases. Abdul et al. [11] showed that the endophytic strain Bacillus subtilis of *Solanum lycopersicum* could secrete IAA, which can significantly increase the biomass of *Solanum lycopersicum*. Plants provide a stable living environment and sufficient nutrients for endophytes. By measuring the endophytic bacterial community of *Lycium barbarum* leaves in different periods, Gou et al. [12] found that the α diversity and richness were the highest in young leaves, that it decreased with the growth of the leaves, and that it reached its lowest in old leaves. However, the composition and structure of endophytic microbial communities have differed in different periods [13], different organs [14], and different varieties of plants [15]. There is great significance in revealing their biological characteristics and disease control properties by exploring the differences in endophytes between dioecious plants. Various secondary metabolites are produced in leaf growth, and plant leaves become an essential source of plant bioactive substances in interaction with microorganisms. Rattanawiwatpong et al. [16] showed that raspberry leaves have vigorous antioxidant activity, and Staszowska-Karkut et al. [17] found that raspberry leaves are rich in phenolic compounds with high development value. Rong et al. [18] found that the chlorogenic acid in *Arctium lappa* leaves has a pan-antibacterial effect. Analyzing the different metabolites found in dioecious plants can reveal specific metabolites and provide a reference for leaf development and utilization.

*Idesia polycarpa* Maxim (Flacourtiaceae) is a deciduous broad-leafed tree. This species is excellent for landscaping because of its unique characteristics, i.e., it is tall, has a straight trunk, and produces bright fruit. [19]. This woody oil tree species has a high development value [20] due to the high oil content in its fruit (77% unsaturated fatty acid content and 62.9% linoleic acid content). In addition, it has the potential to be useful in the preparation of biodiesel [21]. The current state of research on *Idesia polycarpa* focuses mainly on seedling breeding, physiological and biochemical studies, and oil extraction [22]. The lack of reports on the growth differences between male and female plants make such studies even more necessary in this dioecious species.

In this study, we attempted to determine the changes in leaf nutrients, leaf metabolite content, and the differences in the endophytic bacteria community composition between male and female plants in different growth periods. We hypothesized that: (1) Females allocate more resources to reproduction than males. Therefore, the leaves of female plants will accumulate more nutrients and carbohydrates. (2) In our daily observations, males tend to be more resilient than females. Therefore, the endophyte community in male leaves will be more abundant, and the accumulation of stress-resistant substances will be greater.

## 2. Materials and Methods

### 2.1. Study Area

This study was undertaken at the experimental research station (112°42′114°14′ E, and 34°16′34°58′ N) of the College of Forestry, Henan Agricultural University, Zhengzhou, Henan Province, China, in 2021. The mean annual temperature of this site is 14.2 °C, the frost-free period is 215 days, the mean annual precipitation of 650.1 mm, the annual sunshine hours are about 2400 h, and the soil is slightly alkaline sandy loam.

### 2.2. Sample Acquisition

In March 2021, six seven-year-old female and male plants with average growth and no apparent diseases and pests were selected. In May (flowering period), July (fruit matter accumulation period), and October (fruit ripening period), 20 intact mature leaves (each period) were collected from the east, west, north, and south side of the crown and brought back to the laboratory in an ice box. They were fixed at 105 °C for 30 min and dried at 80 °C until the sample weight was constant. The samples were then crushed using a pulverizer, passed through a 100-mesh sieve, and sealed for nutrient analysis. A portion of the sample was frozen in liquid nitrogen before being stored in a −80 °C ultra-low temperature refrigerator to determine the endophytic bacteria and metabolites.

### 2.3. Determination of Leaf Nutrients and Analysis

The leaf nutrients were measured using three samples for each parameter (with three replicates). The leaf pH was measured using an acidometer (LC-PH-3S, Shanghai LiChen Bangxi Instrument Equipment Co., Ltd., Shanghai, China). The total carbon (TC) and total nitrogen (TN) contents were measured using an automatic elemental analyzer (Euro Vector EA3000, Shanghai Wolong Instrument Co., Ltd., Shanghai, China). The total phosphorus (TP) content was determined using the molybdenum antimony anti-colorimetric method, and the total potassium (TK) content was determined using the flame photometer method [23]. Statistical analysis was performed using IBM SPSS v. 26 (IBM Corp., Armonk, NY, USA), and Origin 2017 (www.OriginLab.com (accessed on 30 November 2022)) was used to draw a histogram of nutrient content.

### 2.4. Determination of Endophytic Bacteria in Leaves

#### 2.4.1. Total DNA Extraction, PCR Amplification, and Sequencing

The total DNA was extracted according to the instructions of the E.Z.N.A.^®^ soil kit (Omega Bio-Tek, Norcross, GA, USA). The DNA concentration and purity were detected using NanoDrop2000, and the DNA extraction quality was detected using 1% agarose gel electrophoresis. The V3–V4 region of the bacterial 16S rRNA gene was amplified by PCR using primers 338F (5′-ACTCCTACGGGAGGCAGCAG-3′) and 806R (5′-GGACTACHVGGGTWTCTAAT-3′). The ITS1-ITS2 region of the fungi was amplified by PCR using primers ITS5-1737F (5′-GGAAGTAAAAGTCGTAACAAGG-3′) and ITS2-2043R (5′-GCTGCGTTCTTCATCGATGC-3′). PCR reaction conditions: 95 °C pre-denaturation 3 min, 27 cycles (95 °C denaturation 30 s, 55 °C annealing 30 s, 72 °C extension 30 s), 72 °C extensions 10 min. The amplified products were purified, and a library was constructed. After the library was qualified, it was sequenced using Illumina’s Miseq PE300 platform. The DNA extraction, PCR amplification, and sequencing were entrusted to Wekemo Tech Group Co., Ltd., Shenzhen, China.

#### 2.4.2. Diversity Analysis of Endophytic Bacteria

The sequencing results were subjected to quality control and denoising using the QIIME2 plug-in to obtain valid data. According to the similarity, the sequence was clustered into the operational taxonomic unit (OTU), and species annotation was performed. The alpha diversity index was calculated using QIIME software. LDA (linear discriminant analysis) was performed on each sample using LEfSe software.

### 2.5. Determination of Metabolites in Leaves

#### 2.5.1. Metabolite Extraction

The leaf samples were placed in a freeze-dryer vacuum, freeze-dried, and ground into a fine powder. Next, 100 mg of the powder was dissolved in 1.2 mL of 70% methanol extract. Vortexing was performed for 30 s every 30 min a total of 6 times, and the material was then placed in a 4 °C refrigerator overnight. The supernatant was centrifuged, filtered, and stored in the sample for UPLC–MS/MS analysis.

#### 2.5.2. The UPLC–MS/MS Analysis

Liquid chromatographic conditions: Agilent SB-C18 1.8 um, 2.1 mm × 100 mm; mobile phase: ultra-pure water (with 0.1% formic acid) for phase A and acetonitrile (with 0.1% formic acid) for phase B; elution gradient: 5% for phase B at 0.00 min, linearly increasing to 95% for phase B at 9.00 min and maintaining at 95% for 1 min. The B-phase ratio decreased to 5% at 10.00–11.10 min and was equilibrated at 5% for 14 min; flow rate: 0.35 mL/min; column temperature: 40 °C; injection: 4 uL.

Mass spectrometry conditions: electrospray ionization (ESI) source for bulk data acquisition with the following operating parameters: the ion source, turbo spray: source temperature 550 °C. Ion spray voltage (IS) positive ion mode 5500 V/negative ion mode −4500 V; ion source gas I (GSI), gas II (GSII), and curtain gas (CUR) were set to 50 psi, 60 psi, and 25 psi, respectively, and collision-induced ionization parameters were set at high. Instrument tuning and mass calibration were performed in triple quadrupole (QQQ) and linear ion trap (LIT) modes with 10 and 100 μmol/L polypropylene glycol solutions. QQQ scans were performed using multiple reaction detection (MRM) mode with collisional nitrogen set to medium. In QQQ, each ion pair was scanned for detection according to the optimized declustering voltage (DP) and collision energy (CE).

### 2.6. Data Analysis

Based on the self-built database MWDB (metware database) of Maiwei Metabolic Co., Ltd., the secondary spectrum information and the metabolites were quantified using the multi-reaction monitoring mode of triple quadrupole mass spectrometry. The metabolites of the different samples were compared and analyzed, and the identified metabolites were analyzed using a multivariate statistical analysis to explore the metabolic characteristics of the different samples preliminarily. According to the variable importance projection (VIP) score obtained by an orthogonal partial least squares discriminant analysis (OPLS-DA), the metabolites with VIP ≥ 1, fold change ≥ 2, or fold change ≤ 0.5 were defined as significantly changed metabolites (SCMs). In addition, the corresponding differential metabolites were submitted to the KEGG (Kyoto Encyclopedia of Genes and Genomes) database website, and R software (V4.1.0) was used to draw the heat map of the correlation between the differential metabolites and the leaf nutrients.

## 3. Results

### 3.1. Leaf Nutrient Characteristics in Different Periods

The characteristics of the nutrient content in the leaves at the flowering, fruit matter accumulation, and fruit repining stages were not significantly different. The pH values in the leaves during the reproductive development stages did not differ, and the pH variation range was 5.61–6.18. In addition, there were no significant differences in TC values and TP contents between the male and female plants. The TC content was higher and TP content was lower in female leaves than in male leaves. The TN and TK contents in the leaves of the female plants were higher than in those of the male plants in different periods, and the TN content at the flowering stage was significantly higher than that of the male plants. At the fruit developing stage, TK content was considerably higher in the female plants than in the male plants (Table 1).

### 3.2. Differences of Endophytes in Leaves at Different Stages

#### 3.2.1. OTU Distribution and Alpha Diversity

**Figure 1:**
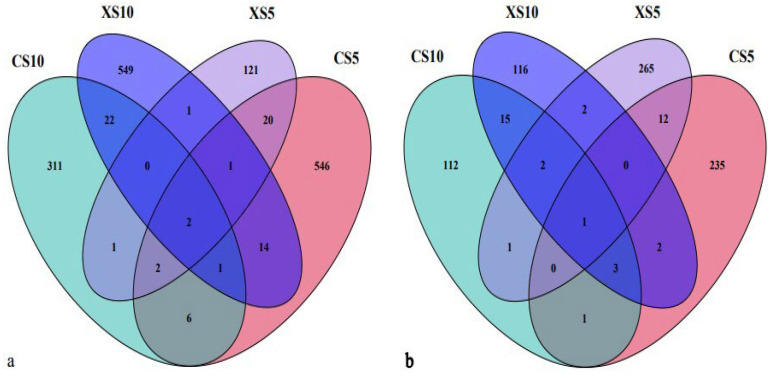
OTU Venn diagram of endophytes in *Idesia polycarpa* leaves. (**a**) Bacteria and (**b**) fungi. Abbreviations: CS10, leaves of the female plants in October; XS10, leaves of the male plants in October; XS5, leaves of the male plants in May; CS5, leaves of the female plants in May.

After high-throughput sequencing, at the 97% similarity classification level, 1597 bacterial OTUs were annotated (Figure 1a). There were 25 OTUs in the leaves of the male and female plants at the flowering stage and 24 OTUs at the fruit ripening stage. The number of unique OTUs in the leaves of the male and female plants in each period was XS10 > CS5 > CS10 > XS5.

A total of 767 fungal OTUs were annotated (Figure 1b). There were 13 OTUs in the leaves of the male and female plants at the flowering stage and 21 OTUs at the fruit maturity stage. The number of unique OTUs in the leaves of the male and female plants in each period was XS5 > CS5 > XS10 > CS10. As a result, it was found that the endophytic composition in the leaves of the male and female plants changed significantly over time.

**Figure 2:**
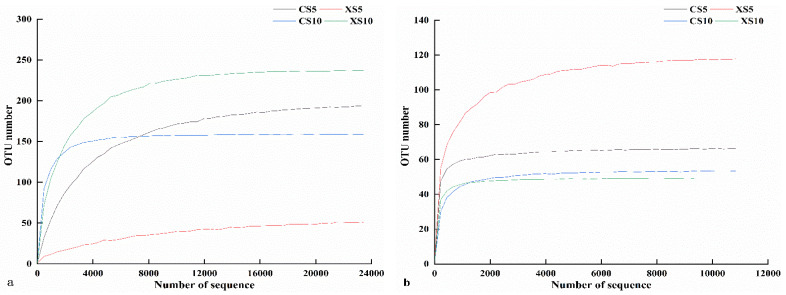
Dilution curve of endophytes in *Idesia polycarpa* leaves. (**a**) Bacteria and (**b**) fungi. Abbreviations: CS5, leaves of the female plants in May; XS5, leaves of the male plants in May; CS10, leaves of the female plants in October; XS10, leaves of the male plants in October.

In the alpha diversity comparison, “Goods coverage” refers to the coverage of each sample library; the higher the value, the lower the probability that the sequence in the sample is not measured. “ACE richness” indicates the flora’s richness; the smaller the value, the lower the richness. “Shannon and Simpson” indicates the flora’s diversity; the smaller the value, the lower the community diversity. The sparse curve of the OTU richness of endophytic bacteria in the leaves of the male and female plants in different periods showed that the number of OTUs of bacteria and fungi in the leaves tended to be stable with the increase in sequencing depth (Figure 2). In addition, the coverage of bacteria and fungi was above 99% (goods coverage), indicating that the sequencing depth of the endophytic bacteria and fungi in the leaves of *Idesia polycarpa* met the requirements of the diversity analysis. There was no significant difference in the ACE index between male and female plants (Table 2). Shannon and Simpson’s indices were significantly different at the stage of fruit development, indicating that the bacterial diversity in the leaves of the male and female plants increased significantly with their growth, but that the richness was not changed. There were no significant differences in the Shannon, Simpson, or ACE indices of fungi in the female leaves (Table 3). In contrast, the Shannon and Simpson of the male leaves showed no significant difference, but the ACE showed a significant difference.

#### 3.2.2. Phylum Level Analysis of Leaf Endophytic Bacteria Community

**Figure 3:**
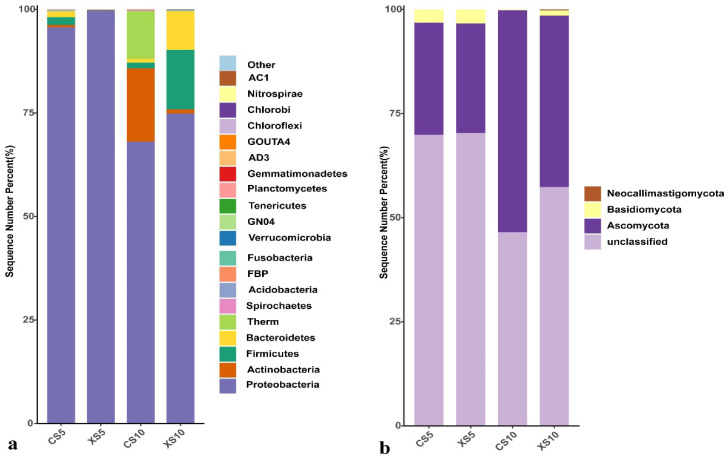
Relative abundance of endophytes in *Idesia polycarpa* leaves at the phylum level. (**a**) Bacteria and (**b**) fungi. Abbreviations: CS5, leaves of the female plants in May; XS5, leaves of the male plants in May; CS10, leaves of the female plants in October; XS10, leaves of the male plants in October.

A total of 20 phyla of endophytic bacteria were detected in the leaves of the male and female *Idesia polycarpa* plants at two growth stages (flowering and fruit ripening) (Figure 3a). Proteobacteria was the dominant group of endophytic bacteria in the male and female leaves, but the abundance was different in the leaves of each period. It was 95.70–99.76% in male and female leaves at the flowering stage and 68.09–74.87% at the fruit ripening stage. The abundance of Firmicutes (1.90%) and Bacteroidetes (1.37%) in the leaves of the female plants at the flowering stage was higher than 1.00%. The abundance of Actinobacteria (17.71%), Thermi (11.54%), and Firmicutes (1.40%) in the leaves of the female plants at the fruit ripening stage was higher than 1%. The abundance of Firmicutes (14.41%), Bacteroidetes (9.32%), and Actinobacteria (1.01%) was higher than 1.00% in the male leaves. Three phyla of endophytic fungi were detected (Figure 3b), among which Ascomycota was the dominant group, accounting for 26.31–26.97% in the leaves of the female and male plants at the flowering stage and 41.15–53.28% at the fruit ripening stage. Moreover, Basidiomycota was more than 1.00% abundant in the leaves of the male plants at both stages (flowering and fruit ripening), but only during the flowering stage in female plants.

#### 3.2.3. Genus Level Analysis of Leaf Endophytic Bacteria Community

**Figure 4:**
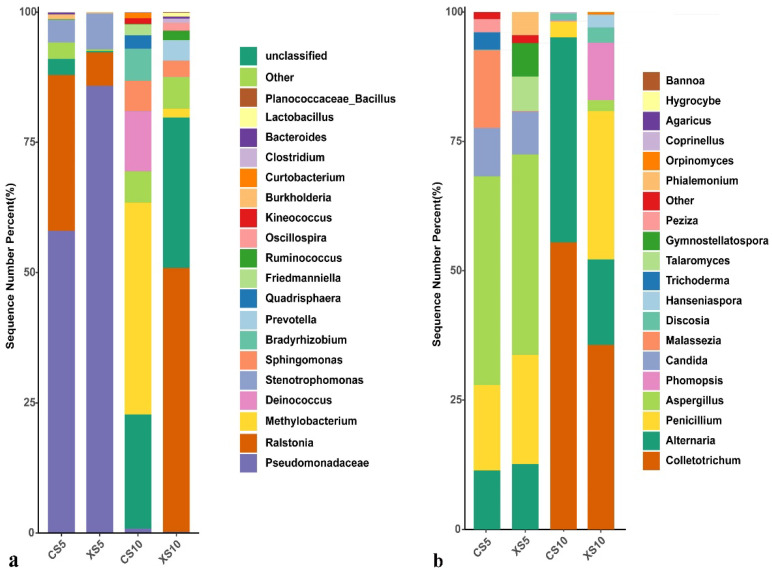
Relative abundance of endophytes in *Idesia polycarpa* leaves at the genus level. (**a**) Bacteria and (**b**) fungi. Abbreviations: CS5, leaves of the female plants in May; XS5, leaves of the male plants in May; CS10, leaves of the female plants in October; XS10, leaves of the male plants in October.

At the genus level, a total of 20 genera of endophytic bacteria were detected in the leaves of the male and female plants at the flowering and fruit ripening stages (Figure 4a). During the flowering period, Pseudomonadaceae (58.01–85.86%) was the most abundant endophytic bacteria in the male and female leaves, followed by Ralstonia (6.38–29.91%) and Stenotrophomonas (4.23–6.90%). The dominant endophytic bacteria in the leaves of the female plants at the fruit ripening stage were Methylobacterium (40.66%), followed by Deinococcus (11.52%) and Bradyrhizobium (6.11%). The dominant endophytic bacteria in the leaves of the male plants were Ralstonia (50.67%), followed by Prevotella (3.91%) and Sphingomonas (3.08%).

In addition, a total of 19 genera of endophytic fungi were detected (Figure 4b). Aspergillus (38.75–40.35%), Penicillium (16.50–21.07%), Alternaria (11.39–12.66%), and Candida (8.29–9.34%) were the most abundant endophytic fungi in the male and female leaves at the flowering stage. Colletotrichum (35.67–55.47%), Alternaria (16.53–39.62%), Penicillium (3.08–28.63%), and Discosia (1.36–2.95%) were the most abundant endophytic fungi in the leaves of the male and female *Idesia polycarpa* plants at the fruit ripening stage.

#### 3.2.4. Difference Analysis of Endophytes in Leaves

**Figure 5:**
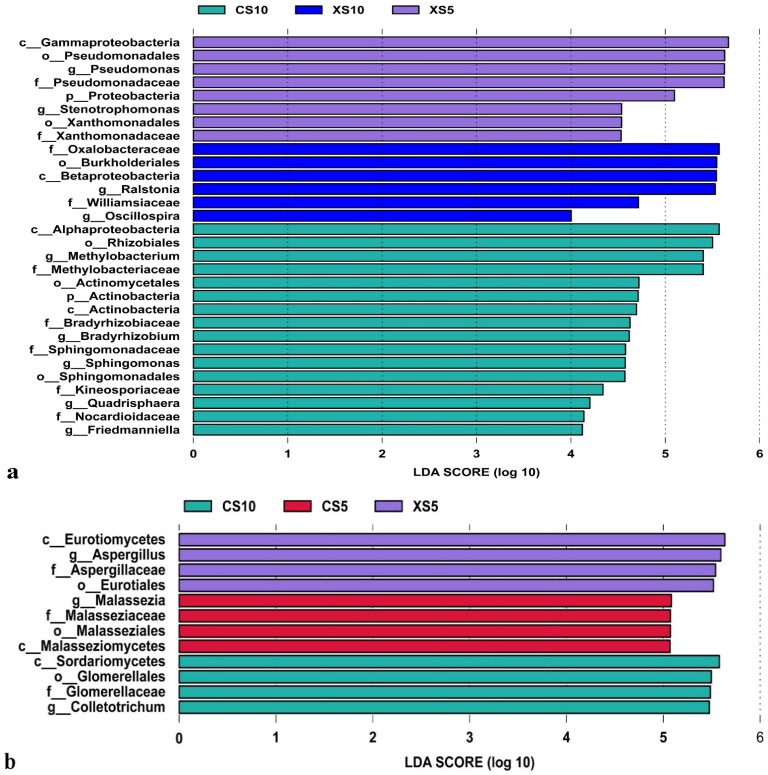
LDA value distribution histogram of endophytes in *Idesia polycarpa* leaves. (**a**) Bacteria and (**b**) fungi. Abbreviations: CS5, leaves of the female plants in May; XS5, leaves of the male plants in May; CS10, leaves of the female plants in October; XS10, leaves of the male plants in October.

To screen out the biological indicator species that can represent the microbial community characteristics of the male and female leaves in each period, LEfSe (LDA effect size) was used to analyze the differences in the abundance of endophytes in the leaves of *Idesia polycarpa* at different growth stages (Figure 5). The LEfSe analysis of the endophytic bacteria showed that there were eight differential indicator species in the XS5 samples, among which Gammaproteobacteria and Pseudomonadales containing Pseudomonas were the most significant groups. In the XS10 samples, the most significant taxonomic units were Oxalobacteraceae, Burkholderiales, and Betaproteobacteria. In the CS10 samples, 16 indicator species were identified, among which Alphaproteobacteria, Rhizobiales, and Methylobacterium accounted for the greatest percentage of significant differences. In the CS5 samples, there was no difference in indicator species.

The LEfSe analysis of endophytic fungi revealed four differential indicator species in the CS5 samples, mainly in Malasseziomycetes. In the XS5 samples, Eurotiomycetes and Aspergillus containing Aspergilllaceae were the most significant genera. The most distinct groups of indicator species in the CS10 samples were Sordariomycetes and Glomerellales containing Glomerellaceae. The above results showed significant differences in the composition of endophytic bacteria in the leaves of the male and female plants at different growth stages. Significant differences were found in the relative abundance of some dominant genera in different fields.

### 3.3. Leaf Metabolite Difference

#### 3.3.1. Sample Principal Component Analysis

**Figure 6:**
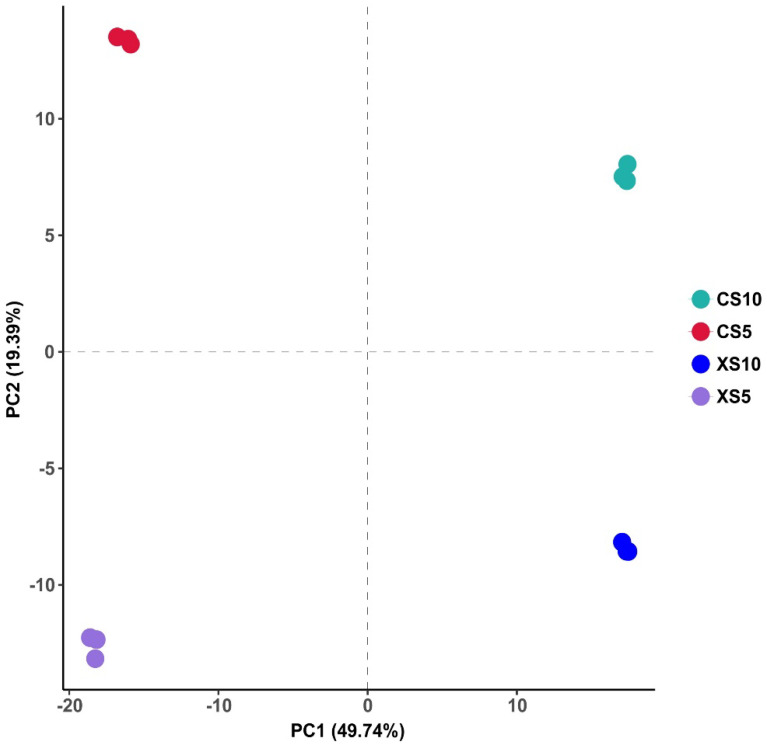
Principal component analysis (PCA) of the leaves. Abbreviations: CS5, leaves of the female plants in May; XS5, leaves of the male plants in May; CS10, leaves of the female plants in October; XS10, leaves of the male plants in October.

A principal component analysis (PCA) was applied to all samples, and the results (Figure 6) showed that the contribution of the first principal component was 49.74%, and that the contribution of the second was 19.39%. Regarding the clustering within the leaf groups of the male and female *Idesia polycarpa* plants at different periods, the distance was small, and the distinction between the sample groups was obvious and distant. On the one hand, the results showed that the repeatability of the determination results was significant. In addition, the leaf metabolites of the male and female plants differed significantly over time.

**Figure 7:**
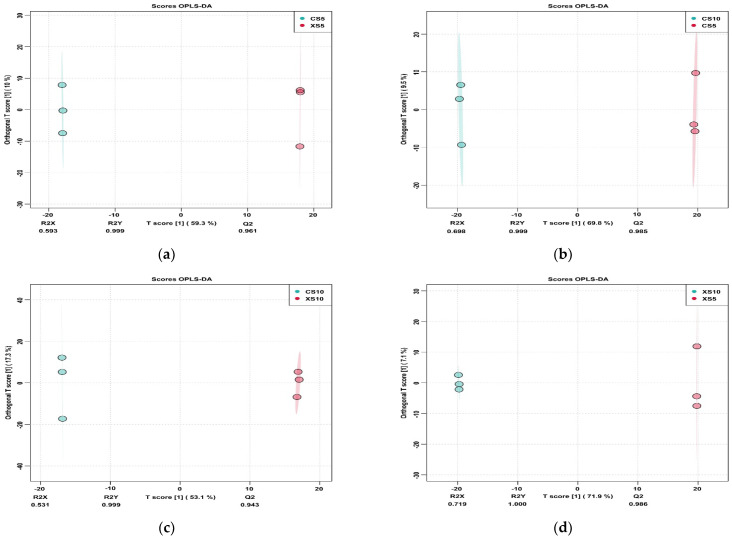
OPLS-DA diagram of differences in metabolites between the two groups of leaves. Abbreviations: (**a**) CS5, leaves of the female plants in May; XS5, leaves of the male plants in May; (**b**) CS10, leaves of the female plants in October; CS5, leaves of the female plants in May; (**c**) CS10, leaves of the female plants in October; XS10, leaves of the male plants in October; (**d**) XS5, leaves of the male plants in May; XS10, leaves of the male plants in October.

The OPLS-DA method was used to analyze the differences in metabolites between the male and female leaves in different periods (Figure 7). The results showed that the separation effect between the male and female leaves was significant in two periods. Model reliability verification showed that the four models are stable, and the results are highly reliable.

#### 3.3.2. Screening of Differential Metabolites in Leaves

A total of 694 metabolites with significant differences were selected from the four comparison groups and were screened based on the *p* value (*p*-value < 0.05) of the t-test and the VIP (VIP > 1) of the OPLS-DA model, and most of them were phenolic acids. Each group produced 87 to 254 significantly different metabolites. The top five significantly different metabolites, ranked in descending order by the VIP values of the OPLS-DA model, are shown in Table 4. The abundance of salireposide in the leaves of the male plants was significantly higher than that in the leaves of the female plants at the flowering stage. However, it became significantly lower than that in the leaves of the female plants in October. Stearidonoyl-glycerol was significantly less abundant in the male and female plants during the fruit ripening stage than in the flowering stage.

#### 3.3.3. Metabolic Pathway Analysis

**Figure 8:**
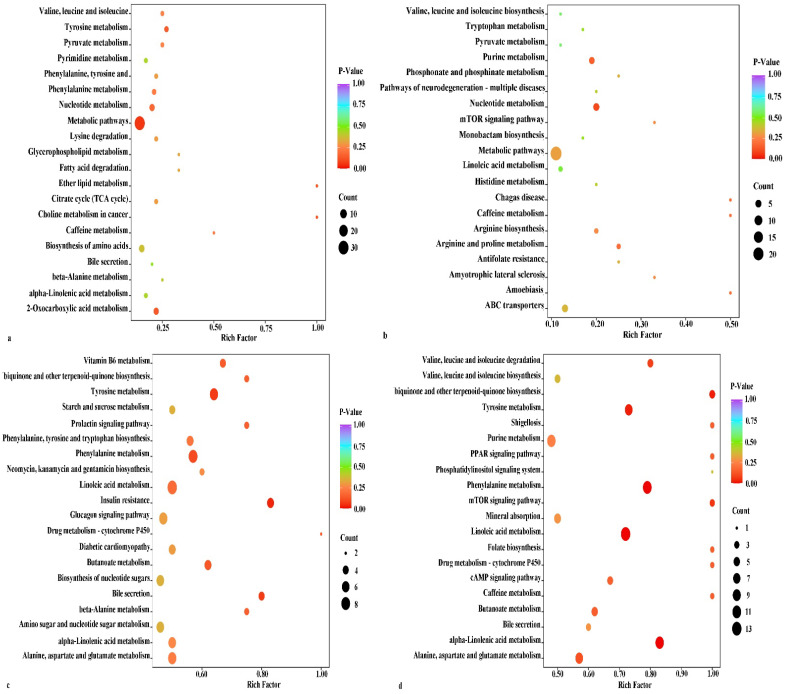
KEGG pathway map for leaf differential metabolites. Abbreviations: (**a**) CS5 vs. XS5, leaves of the female plants in May vs. leaves of the male plants in May; (**b**) CS10 vs. XS10, leaves of the female plants in October vs. leaves of the male plants in October; (**c**) CS5 vs. CS10, leaves of the female plants in May vs. leaves of the female plants in October; (**d**) XS5 vs. XS10, leaves of the male plants in May vs. leaves of the male plants in October.

To determine the mechanism by which the differential metabolites vary from one period to another in the leaves of the male and female *Idesia polycarpa*, we utilized the KEGG database for functional annotation and pathway enrichment analysis. The 108 differential metabolites screened in the CS5 vs. XS5 group were annotated to 34 metabolic pathways (Figure 8a). More metabolites were found in the metabolic pathways, followed by the biosynthesis of amino acids, the nucleotide metabolism, and the 2-oxocarboxylic acid metabolism. In the CS10 vs. XS10 group, the 87 differential metabolites were screened and annotated to 35 metabolic pathways (Figure 8b). More metabolites were concentrated in the metabolic pathways, followed by the ABC transporters and the nucleotide metabolism.

A total of 245 differential metabolites were identified in the CS5 vs. CS10 group and annotated to 128 pathways (Figure 8c). The insulin resistance was significantly enriched (*p* < 0.05). The differential metabolites involved in insulin resistance were D-fuctose6-phosphate*, uridine 5′-diphospho-N-acetylglucosamine, D-glucose 6-phosphate*, D-glucosamine 1-phosphate, and D-glucose*.

A total of 254 differential metabolites screened in the XS5 vs. XS10 group were annotated to 124 pathways (Figure 8d). The alpha-linolenic acid metabolism, phenylalanine metabolism, and linoleic acid metabolism were significantly enriched (*p* < 0.01), with 10, 11, and 13 differential metabolites involved, respectively. The tyrosine metabolism, ubiquinone, and other terpenoid–quinone biosynthesis pathways were significantly enriched (*p* < 0.05), and eight and four differential metabolites were involved, respectively. In addition, a large number of the differential metabolites of the male and female leaves were enriched. The common pathways were the metabolic pathways, the biosynthesis of amino acids, and the nucleotide metabolism.

### 3.4. Correlation Analysis of Leaf Nutrients with the Microbial Community and Differential Metabolites

**Figure 9:**
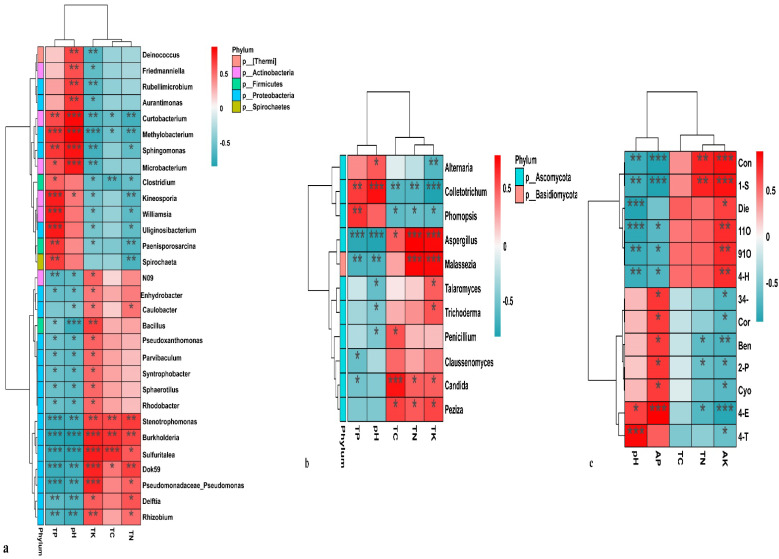
Correlation analysis of leaf nutrients with the microbial community and differential metabolites. (**a**) Bacteria. TP, total phosphorus; TK, total potassium; TC, total carbon, TN, total nitrogen. (**b**) Fungi. TP, total phosphorus; TK, total potassium; TC, total carbon, TN, total nitrogen. (**c**) Metabolites; TP, total phosphorus; TK, total potassium; TC, total carbon, TN, total nitrogen; Cyo, Cyclo(l-phe-l-pro); 2-P, 2-Phenylethyl beta-d-glucopyranoside; Sal, Salireposide; 4-O, 4-*O*-(6′-*O*-glucosyl-p-coumaroyl)-4-hydroxybenzyl alcohol; 4-E, 4-Ethoxyphenol; 3,4-, 3,4-Dimethoxyphenol; 8-H, 8-Hydroxyguanosine; Die, Diethyl phosphate; Cor, Cordycepin (3′-deoxyadenosine); 1-S, 1-Stearidonoyl-glycerol; Con, Coniferaldehyde; Ben, Benzoic acid; 4-T, 4-(3,4,5-Trihydroxybenzoxy)benzoic acid; 11O, 1,18-Octadecanediol; 91O, 9,12-Octadecadien-6-ynoic acid; 4-H, 4-Hydroxy-3-methoxymandelate. The single asterisk (*) mark represents *p* < 0.05 value; double asterisk (**) marks represent *p* < 0.01 value; and triple asterisk (***) marks represent *p* < 0.001 value.

*Idesia polycarpa* leaves exhibit significantly different metabolites and nutrient contents based on the correlation analysis of the top 30 endophytes. The results showed that the TK content in the leaves had the most significant effect on the endophytic bacteria in leaves (Figure 9a), and that these were significantly correlated (*p* < 0.05/0.01/0.001). The pH and total phosphorus content in the leaves had a negative correlation with Proteobacteria and a positive correlation with Actinobacteria. In addition, the total potassium and total nitrogen contents correlated negatively with Proteobacteria and positively with Actinobacteria. There was less correlation between the total carbon content in leaves and the endophytic bacteria. The TK content in the leaves significantly affected the endophytic fungi (Figure 9b) and showed a significant relationship. Colletotrichum was positively correlated with the TC, TN, and TK content in the leaves and negatively correlated with the pH and TP content, while the opposite was true for Aspergillus. The TK content of the leaves significantly affected the content of differential metabolites (Figure 9c). No significant relationship was found between the TC content and the differential metabolites in the leaves. Coniferaldehyde and 1-stearidonoyl-glycerol were positively correlated with TN and TK, and significantly negatively correlated with pH and TP, while the opposite was true for 4-ethoxyphenol. In general, the TK content in the leaves significantly affected the endophytic bacteria and differential metabolites.

## 4. Discussion

### 4.1. Nutrient Contents in Idesia Polycarpa Leaves

The growth and development of plants require nutrients such as carbon, the skeleton element of cells, and nitrogen and phosphorus are essential elements in plant photosynthesis [24]. There is a close correlation between the element characteristics of plant leaves and the basic behavior and functions of plants. The nutrient composition of leaves can also reflect the level of nutrient intake by plants [25]. In this study, there was no significant difference between the male and female plants at different growth stages in terms of TC or TP content. However, the TN content in the leaves of the female plants was significantly higher than that in the leaves of the male plants at the flowering stage. The ratio of carbon to phosphorus and nitrogen to phosphorus in leaves can reflect the ability of a plant to assimilate carbon [26]. The period after flowering is when the plants begin to reproduce. The female plant needs to pay higher reproductive costs than the male plant, which may maintain its consumption by increasing the leaf nitrogen content and enhancing its photosynthesis. Carbon is an important raw material for plant photosynthesis. There was no significant difference in carbon content between the male and female leaves, and the reasons for this need to be explored in more depth. The TK content in the leaves of the female plants was significantly higher than that in the leaves of the male plants at the fruit matter accumulation stage, and similar results were found by Fu Hao et al. [27] in *Zanthoxylum schinifolium*. Potassium can promote the transport of assimilates [28], and thus the accumulation of more potassium in female leaves can promote the accumulation of fruit matter and fruit development.

### 4.2. Endophytes in Idesia Polycarpa Leaves

Leaf endophytes widely exist in various plants and affect plant growth, development, and metabolic activities [29]. This paper used high-throughput sequencing technology to analyze the community characteristics of endophytic microorganisms in the leaves of male and female plants in different periods. The male and female plants produced different diversity indices in the same period and composed fewer common OTUs in their leaves. There were abundant and diverse microbial communities in the leaves of the male and female plants. Further analysis showed that Proteobacteria, Pseudomonadaceae, Ascomycota, and Aspergillus were the dominant bacteria and fungi in the leaves of *Idesia polycarpa*, and these are similar to the endophytic bacteria found in other plants [30]. Previous studies have shown that microorganisms such as Proteobacteria, Pseudomonadaceae, and Aspergillus play an important role in enhancing plant resistance, promoting plant growth, and preventing disease [31]. According to Liu et al. [32], phyllosphere bacteria differed at the genus level between male and female *Fraxinus chinensis*. We found differences in the composition of endophytes in the leaves of the male and female plants at different stages. At the flowering stage, the relative abundance of Ralstonia was higher in female leaves, while the relative abundance of Pseudomonadaceae was higher in male leaves. It is generally believed that Ralstonia is a pathogen that causes plant diseases [33]. Pseudomonadaceae can increase the diversity of microbial communities and enhance plant resistance by improving nutrient use efficiency [34]. During the fruit accumulation period, the relative abundance of Methylobacterium and Deinococcus in the leaves of the female plants was higher. Methylobacterium can secrete cytokinins to stimulate plant growth [35], and Deinococcus has a strong pollution removal ability [36]. The more beneficial endophytes enriched in the leaves of female plants can enhance plant resistance and ensure the normal development of fruit. The relative abundance of Ralstonia and Prevotella in the leaves of male plants was higher. Prevotella can produce antibiotics and enhance the chemical defense ability of leaves [37]. Plants and these endophytes are mutually beneficial. Plant leaves provide a stable environment and nutrients for endophytes, and endophytes help plants grow and resist adverse conditions. In addition, abundant beneficial endophytic bacteria in the leaves of *Idesia polycarpa* provide a good foundation for excavating functional strains.

### 4.3. Metabolites in Idesia polycarpa Leaves

The leaf is the main component and a crucial vegetative organ of plants, and its metabolite data can reflect the physiological characteristics of the whole plant [38]. This paper found 694 metabolites with significant differences from the leaves of male and female plants in different periods. There were 195 significantly different metabolites between the male and female leaves, mainly phenolic acids, indicating that the leaves of the male and female plants meet their own needs and adjust phenolic acids accordingly. Compared with the female plants, the phenolic acid content in the leaves of the male plants increased at the flowering and fruit ripening stages. Rabska Mariola et al. [39] conducted a pot experiment on *Juniperus communis* L and found that under abiotic stress, the concentration of phenolic compounds in the leaves of male and female *Juniperus communis* L plants decreased, and that the concentration of phenolic compounds was higher in the female plants than in the male plants. Zhang et al. [40] confirmed that the decrease in the number of phenolic compounds in the leaves of *Populus yunnanensis* under drought stress was greater in female plants than in male plants. The climate and water might be the reason for the difference in phenolic compounds in the leaves of the male and female plants. Phenolic acids have a variety of physiological functions, such as anti-oxidation, regulation of enzyme activity, and resistance to pathogens [41]. This shows that the resistance of male plants is stronger than that of female plants, which may be related to the fact that female plants use more substances for reproduction. Using the leaves at the flowering stage as a control, the lipid content of the leaves at the fruit ripening stage was observed to decreased significantly. Lipids are crucial for plant energy conversion, carbon storage, signal transduction, and stress responses [42]. This is consistent with the growth habit and environment of *Idesia polycarpa* at the reproductive stage.

### 4.4. Leaf Nutrients with the Microbial Community and Differential Metabolites

It has been found that the dynamic changes in the endophytic bacteria community and metabolite content in plants were affected by many factors, including plant species [43], plant growth, and development stage [44]. Leaves are the environment for the survival of endophytes and the basis for the existence of metabolites. Endophyte composition and metabolite content are closely related to the nutritional content of the plant. Chen et al. [45] found that nitrogen, phosphorus, and potassium in the environment had a significant positive effect on the relative abundance of *Sebacina* sp. Zhang et al. [46] believed that an appropriate amount of phosphate fertilizer could increase the content of chlorogenic acid and total flavonoids in Chrysanthemi indici Flos. As the basic raw material of organic matter, carbon is closely related to the synthesis and metabolism of organic matter. However, this paper has demonstrated no significant correlation between the total carbon content and the differential metabolites in the leaves of *Idesia polycarpa* plants, indicating the efficient utilization of carbon in the leaves of the male and female plants. The TK content in leaves was significantly related to the abundance of most endophytic bacteria and the content of metabolites. This indicated that the potassium content in the leaves played an essential role in constructing the internal environment of leaves, which was also consistent with the view that potassium could improve plant resistance. In addition, the total phosphorus content in the leaves had a positive correlation with Actinobacteria and phenolic acids and a negative correlation with Ascomycota. Phosphate can promote plant growth and an increase in dry-weight biomass [47], and one of the ways it does this might be by increasing the abundance of beneficial endophytes and enhancing the level of metabolism. In production, the richness and diversity of endophytes, the contents of beneficial microorganisms, and the resistant substances in the leaves of *Idesia polycarpa* could be increased by using microbial potassium and phosphorus fertilizers, thus promoting the growth of the *Idesia polycarpa* species.

## 5. Conclusions

In this study, the nutrient characteristics, endophytes, and metabolites were analyzed in the leaves of male and female Idesia polycarpa at different reproductive stages. The results revealed differences in the absorption of nitrogen and potassium between the male and female plants. We identified significantly different metabolites in the leaves of the female and male plants based on their diversity and endophyte and phenolic acid compositions. Through correlation, it was found that the leaf endophytes and significantly different metabolites were most affected by the content of TK and TP in the leaves, but were less affected by TC and TN content. For future use, this study provides a reference for separating endophytic bacteria, excavating functional strains, developing and utilizing leaves, and managing production.

## Figures and Tables

**Table 1:** The nutrient characteristics of *Idesia polycarpa* leaves in different reproductive stages. The lowercase letters indicate significant differences. Abbreviations: CS, Female; XS, Male.

Period	Sexuality	pH	Total Carbon g·kg^−1^	Total Nitrogen g·kg^−1^	Total Phosphorus g·kg^−1^	Total Potassium g·kg^−1^
May	CS	5.70 ± 0.48 a	551.17 ± 43.57 a	34.15 ± 0.63 a	4.60 ± 0.17 a	10.78 ± 1.10 a
	XS	5.73 ± 0.53 a	521.20 ± 13.67 a	29.32 ± 1.89 b	5.13 ± 0.1 a	8.80 ± 1.34 a
July	CS	5.67 ± 0.04 a	446.13 ± 3.53 a	18.40 ± 1.98 a	4.37 ± 0.41 a	9.90 ± 2.98 a
	XS	5.72 ± 0.09 a	439.39 ± 4.55 a	17.62 ± 2.14 a	3.17 ± 0.33 a	3.08 ± 0.58 b
October	CS	6.15 ± 0.03 a	489.20 ± 12.53 a	24.84 ± 2.57 a	6.82 ± 0.42 a	5.24 ± 0.03 a
	XS	5.97 ± 0.05 b	492.86 ± 2.89 a	23.83 ± 2.21 a	7.96 ± 0.54 a	4.56 ± 0.39 a

**Table 2:** Leaf bacterial α diversity index. Different lowercase letters in the same column indicate significant differences. Abbreviations: CS5, leaves of the female plants in May; XS5, leaves of the male plants in May; CS10, leaves of female plants in October; XS10, leaves of the male plants in October.

Samples	Goods Coverage	Shannon	Simpson	ACE Richness
CS5	1.00 ± 0.00 a	1.44 ± 0.6 bc	0.30 ± 0.06 c	197.38 ± 144.84 a
XS5	1.00 ± 0.00 a	0.93 ± 0.06 c	0.27 ± 0.01 c	55 ± 13.05 a
CS10	1.00 ± 0.00 a	5.18 ± 0.17 a	0.92 ± 0.01 a	157 ± 6.03 a
XS10	1.00 ± 0.00 a	3.51 ± 1.23 ab	0.69 ± 0.12 b	236.67 ± 78.56 a

**Table 3:** Leaf fungal α diversity index. Different lowercase letters in the same column indicate significant differences. Abbreviations: CS5, leaves of the female plants in May; XS5, leaves of the male plants in May; CS10, leaves of female plants in October; XS10, leaves of the male plants in October.

Samples	Goods Coverage	Shannon	Simpson	ACE Richness
CS5	1.00 ± 0.00 a	5.12 ± 0.25 a	0.95 ± 0.01 a	67.33 ± 1.45 ab
XS5	1.00 ± 0.00 a	5.14 ± 0.57 a	0.95 ± 0.02 a	105.33 ± 25.67 a
CS10	1.00 ± 0.00 a	3.85 ± 0.59 a	0.82 ± 0.09 a	67 ± 13.53 ab
XS10	1.00 ± 0.00 a	4.53 ± 0.14 a	0.92 ± 0.02 a	49.33 ± 3.33 b

**Table 4:** Top five differential metabolites according to their VIP value in the four comparison groups.

Comparison Group	Total Number of Differential Metabolites	VIP Value Top Five Metabolites	Log2 FC	*p*-Value	VIP	Metabolite Types
CS5 vs. XS5	108	Cyclo (l-phe-l-pro)	9.671	0.000	1.288	Amino acids and derivatives
		2-Phenylethyl beta-d-glucopyranoside	13.186	0.001	1.288	Phenolic acids
		Salireposide	10.385	0.002	1.288	Phenolic acids
		4-*O*-(6′-*O*-glucosyl-p-coumaroyl)-4-hydroxybenzyl alcohol	3.356	0.000	1.288	Phenolic acids
		4-Ethoxyphenol	11.342	0.005	1.288	Phenolic acids
CS10 vs. XS10	87	3,4-Dimethoxyphenol	10.558	0.004	1.346	Phenolic acids
		8-Hydroxyguanosine	11.931	0.004	1.346	Nucleotides and derivatives
		Salireposide	−10.336	0.005	1.346	Phenolic acids
		Diethyl phosphate	9.431	0.004	1.346	Organic acids
		Cordycepin (3′-Deoxyadenosine)	10.013	0.005	1.346	Nucleotides and derivatives
CS5 vs. CS10	245	1-Stearidonoyl-glycerol	−15.663	0.000	1.175	Lipids
		Cyclo(l-phe-l-pro)	8.051	0.000	1.175	Amino acids and derivatives
		4-Ethoxyphenol	12.138	0.001	1.175	Phenolic acids
		Coniferaldehyde	−12.447	0.001	1.175	Phenolic acids
		Benzoic acid	11.595	0.001	1.175	Phenolic acids
XS5 vs. XS10	254	1-Stearidonoyl-glycerol	−12.498	0.000	1.166	Lipids
		4-(3,4,5-Trihydroxybenzoxy) benzoic acid	3.737	0.000	1.166	Phenolic acids
		1,18-Octadecanediol	−10.018	0.001	1.166	Lipids
		9,12-Octadecadien-6-ynoic acid	−11.759	0.002	1.166	Lipids
		4-Hydroxy-3-methoxymandelate	−14.586	0.003	1.166	Organic acids

## Data Availability

The data sets generated and/or analyzed during the current study are available from the corresponding author upon reasonable request.
